# Experimentally increasing sedentary behavior results in decreased life satisfaction

**DOI:** 10.15171/hpp.2017.16

**Published:** 2017-03-05

**Authors:** Meghan K. Edwards, Paul D. Loprinzi

**Affiliations:** Department of Health, Exercise Science and Recreation Management, Physical Activity Epidemiology Laboratory, Exercise Psychology Laboratory, The University of Mississippi, University, MS, USA

**Keywords:** Physical activity, Quality of life, Subjective well-being, Randomized control intervention

## Abstract

**Background: ** No study has experimentally manipulated sedentary behavior and evaluated its effect on life satisfaction. Thus, the purpose of this study was to evaluate the effects of a free-living, sedentary behavior-inducing randomized controlled intervention on life satisfaction.

**Methods:** Active, young adults between the ages of 18-35 were recruited and randomly assigned into a sedentary behavior intervention group (n = 26) or a control group (n = 13). The intervention group participants were instructed to eliminate all exercise and restrict daily steps (as measured via pedometry) to 5000 or less per day for one week. The control group was instructed to maintain regular levels of exercise and other physical activity for one week. Both groups completed the Satisfaction with Life Scale (SWLS) pre-intervention and immediately post-intervention.

**Results:** There was a significant group x time interaction (F = 32.75, P < 0.001), with post-hoc contrast tests indicating decreased SWLS score (indicating lower levels of life satisfaction) in the intervention group during Visit 2 (post-intervention) compared with Visit 1 (pre-intervention); this corresponded with a mean absolute (Visit 2 minus Visit 1) change of -8.58 (95% CI: -5.91, -11.24) for SWLS scores in the intervention group (31.1% reduction).

**Conclusion:** A one-week sedentary behavior-inducing intervention may negatively impact life satisfaction in an active, young adult population. Regular physical activity may be imperative in avoiding negative life satisfaction-related consequences.

## Introduction

Quality of life is a term often used to describe the overall human experience. Most individuals, communities, and societies share a common goal of either improving or maintaining desirable levels of quality of life, thus engaging in a positive overall human experience.^1^ Previous research has suggested that there are two major components of quality of life.^1^ The first component involves the examination of various social and economic indicators that ultimately aim to demonstrate how well an individual’s needs are being met.^2^ An initial theory of basic human needs (e.g., physiological needs, safety needs, and love needs) required for the process of motivation was formed in the 1940s,^3^ which arguably served as a catalyst to the study of human needs and a point of reference for future needs-related work (e.g., human needs as they relate to quality of life). As mentioned previously, quality of life is believed to be in part determined by the degree to which needs (e.g., subsistence, security, affection, understanding, creativity, leisure, identity, and freedom^1^) are being met. The measurement of needs being met has been described as relatively (when compared to the second quality of life component) objective in nature (i.e., the needs are either being met, or they are not).^1^

The second type of quality of life assessment is a general construct referred to as *subjective well-being* (SWB), which evaluates outcomes that are subjective in nature (e.g., self-reported happiness, pleasure, and fulfillment).^4^ Previous work on SWB has identified two main components or types. The first component of SWB is affective in nature (i.e. having to do with mood states), typically further distinguished as either pleasant affect or unpleasant affect.^5^ The second type of SWB is cognitive in nature and is referred to as life satisfaction.^6^ Previous work regarding SWB has focused mainly on the affect-related parameters, with less emphasis on the life satisfaction component of SWB.^7^

Life satisfaction, the outcome variable of interest in our study, is said to involve a conscious judgmental process in which individuals employ a unique set of criteria to assess the quality of their own lives.^8^ Global judgment of life satisfaction is predicted to depend upon the comparison of one’s life circumstances to their unique standards.^7^ Much research regarding life satisfaction supports the opinion that factors influencing this outcome may have either a top-down or a bottom-up effect.^9^ Top-down influences are time-invariant, trait-like (e.g., mental health, body mass index [BMI], overall physical activity, personality, self-esteem, sex) and allow for the evaluation of between-person differences and similarities.^10^ Bottom-up influences vary with time, are state-like (e.g., fatigue, self-esteem mental health, daily physical activity) and allow for the evaluation of within-person differences and similarities.^10^ Some factors, such as physical activity, have been demonstrated to have both top-down and bottom-up positive associations with life satisfaction and have thus received considerable recent attention in research aiming to distinguish which effects (i.e., top-down or bottom-up) may have the most significant influences.^10^ Also contributing to our knowledge of the physical activity-life satisfaction relationship are prospective studies on elderly populations, which have demonstrated the positive effects on life satisfaction as related to regular physical activity.^11,12^ Physical activity is believed to indirectly enhance one’s life satisfaction via influences on affect, physical self-worth, self-efficacy, and mental health.^11^

While it is important to understand how physical activity may influence one’s life satisfaction, emerging research suggests that regardless physical activity levels, sedentary behavior is associated with a number of negative health outcomes.^13^ Recent epidemiological work has demonstrated a negative association between objectively measured sedentary behavior and life satisfaction, independent of (objectively measured) physical activity levels.^14^ However, no studies (to our knowledge) have utilized an experimentally designed sedentary behavior intervention to draw conclusions upon.

The purpose of this study was to add to existing knowledge on the association between sedentary behavior and life satisfaction via a randomized, controlled sedentary behavior intervention study. Among an “active” sample, we assessed life satisfaction to determine if a sedentary behavior intervention (i.e., minimizing physical activity and increasing sedentary behavior) significantly altered this outcome. We hypothesized active individuals whose sedentary behavior was increased for one week would report a lower post-intervention life satisfaction. In addition, we hypothesized that life satisfaction scores would improve after normal activity was resumed (i.e. returned to baseline levels). This hypothesis is plausible because, as mentioned previously, observational-based research has demonstrated a positive relationship between physical activity and life satisfaction.^10,14^ Thus, it is reasonable to suggest that life satisfaction may worsen if sedentary behavior is increased. This approach may provide the strongest evidence of a potential cause-and-effect relationship between sedentary behavior and life satisfaction.

## Materials and Methods

### Recruitment 

To participate in the study, participants had to be between 18 and 35 years old, be active, speak English, and provide written informed consent. Participants with inadequate levels of physical activity (described below) in the week of accelerometer data collection prior to the intervention were excluded from the study. The authors’ institutional review board approved all study procedures prior to the start of data collection.

The recruitment goal for this study was 30-40 participants with a minimum of n = 22 in the intervention group. This goal was based on pilot data^15^ demonstrating that, among a sample of 29 participants who had similar demographic characteristics to the participants in the present study, prospective changes in sedentary behavior were associated with depression symptomology. A student researcher at the authors’ institution used a non-probability convenience sampling approach to recruit all participants. The final sample size was N = 39; using a 2:1 sample size ratio for intervention and control participants,^16^ 26 participants were randomly assigned into the sedentary behavior intervention group and 13 participants were randomly assigned into the control group. A 2:1 sample size ratio was used due to considerations related to study resources. Of note, experimental-to-control ratios of 2:1 do not substantively reduce statistical power, and unequal allocation (if performed randomly) still results in equivalent groups in terms of equal distribution of confounding parameters.

### Visit protocol

A detailed description of the protocol for the study visits can be found elsewhere.^17^ Briefly, we recruited only active individuals who attained a minimum of 150-minutes/week of moderate-to-vigorous physical activity. Individuals wore an accelerometer for one week after completing a self-reported assessment of physical activity so that we could objectively confirm their activity level. Individuals were randomly assigned to an intervention or a control group. The intervention group was asked to omit all exercise for one week, and to reduce their steps below 5000/day for a 1-week duration. The control group was asked to continue with their normal physical activity/exercise routines. At the conclusion of the sedentary-intervention week, the control group was then finished with the study, whereas the intervention group was asked to resume normal physical activity/exercise levels for one week. Satisfaction with life was assessed at baseline and post-intervention week in both groups. The intervention group had a third assessment time point, following the 1-week of normal physical activity/exercise.

### Measures

Physical activity

The initial assessment of physical activity levels was assessed via the IPAQ-SF,^18^ with objective confirmation of this activity measured using ActiGraph GT9X accelerometers. Notably, we discuss the psychometric properties of both of these measures elsewhere.^19^

Satisfaction with life

To assess life satisfaction, we utilized the Satisfaction with Life Scale (SWLS).^20^ This survey includes five statements (e.g., “The conditions of my life are excellent.”), to which respondents must rate how much they agree with, on a 7-point Likert scale (1 = strongly disagree and 7 = strongly agree).^20^ The SWLS items are global in nature, which gives respondents the opportunity to weigh the various domains of their lives in terms of their own values. It is thus considered to provide a global judgment of life satisfaction.^7^ In a study of college-aged students (with similar ages as individuals in our current study), the SWLS was found to have good test-retest reliability (0.82). The SWLS has also been demonstrated to have adequate levels of internal consistency (α = 0.61-0.81) and convergent validity when compared with the Life Styles Inventory (*r* = 0.46).^20^

Internal consistency (Cronbach’s alpha) was calculated to be 0.80 during Visit 1 within the intervention group and 0.93 during Visit 1 within the control group. During Visit 2, internal consistency was 0.93 within the intervention group, and 0.92 within the control group. Internal consistency for the intervention group during Visit 3 was 0.88.

### Statistical analysis

Analysis was computed using SPSS software (version 22.0) and Stata software (version 12.0). Demographic differences between the two groups at baseline were compared via independent *t* tests for any continuous data (age, BMI, and mean moderate-to-vigorous physical activity [MVPA]) and via chi-square tests for any nominal data (education status, race/ethnicity and gender). To examine the effects of the sedentary behavior intervention on life satisfaction, a split-plot 2 × 2 analysis of variance (ANOVA) was computed in SPSS, with SWLS scores as the outcome variable. For the split-plot analysis, condition was the between-subject variable, and time was the within-subject variable. We utilized a 2 × 2 split-plot ANOVA due to the fact that the control group met for one less visit than the intervention group. No assumptions for this split-plot 2 × 2 ANOVA were violated. Following the split-plot ANOVA (given a significant interaction), a paired *t* test (simple effect) was conducted in Stata to examine differences in SWLS between the second and third visit. Given the relatively small sample size, additional sensitivity analyses were conducted using the Wilcoxon signed rank sum test; results were similar to the parametric paired-samples *t* test (data not shown). Effect size estimates were calculated to estimate strengths of associations (Ƞ^2^_p_; partial eta-squared). The Ƞ^2^_p_ estimate was calculated using formula #13 in the reference by Lakens.^21^ Due to the relatively small sample size of the present study, the *corrected* Ƞ^2^_p_ (partial omega squared, ω^2^_p_) was also calculated using formula #15 in Lankens.^21^ Additionally, Cohen’s d values were calculated to assess mean difference effect sizes, both between and within groups. The between group mean difference effect size was calculated using Formula #1 in Lakens whereas the within group mean difference effect sizes were calculated using Formula #7 in Lakens.^21^ A two-tailed nominal α of 0.05 was set as the level for statistical significance.

## Results

Descriptive characteristics of the study sample are shown in Table 1. The sample sizes of the two groups were n=26 in the intervention and n = 13 in the control. The mean age of the intervention group was 21.69 (SD = 2.71); 38% of the participants were male compared to the control group, which had a mean age of 22.08 (SD = 2.75) years and 46% males. Table 1 displays that there were no statistically significant differences between the two groups with regards to any of the collected demographic characteristics. As such, these parameters were not included as covariates in our analysis.

**Figure 1:**
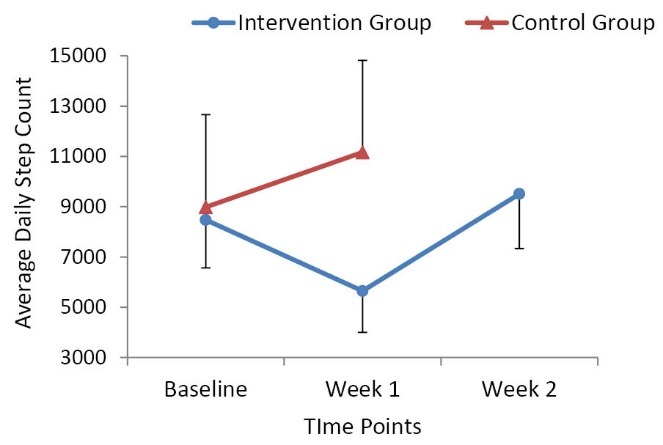
Intervention group vs. control group average daily step counts over time.
Standard deviations are included as the error bars.

Step counts decreased significantly (*P *< 0.0001) after the 1-week sedentary behavior-inducing intervention. In particular, steps/day decreased from 8475.13 (SD = 1902.96) to 5648.60 (SD = 1646.37). In intervention group, after resuming normal physical activity (week 2), steps were significantly higher (*P *< 0.0001) than in week 1 (9508.35 [SD = 2172.80] vs. 5648.60 [SD = 1646.37]) but were not significantly different (*P *= 0.06) than baseline steps (9508.35 [SD = 2172.80] vs. 8475.13 [SD = 1902.96]). These findings demonstrate that the intervention group significantly reduced their steps from baseline to week 1, with physical activity returning to near baseline levels in week 2. In the control group we also saw a significant increase in the number of steps from baseline to week 1 (8983.60 [SD = 3679.83] vs. 11 165.73 [SD = 3654.08], *P *= 0.03). Sufficient pedometry wear time was reported for all participants with 14.25 h/d during week 1, the initial intervention week (N = 39) and 14.93 h/d during week 2, the 1-week of resumed physical activity following the intervention (n = 26). Figure 1 contains a graphical display of the mean daily steps over time within each group.

**Figure 2:**
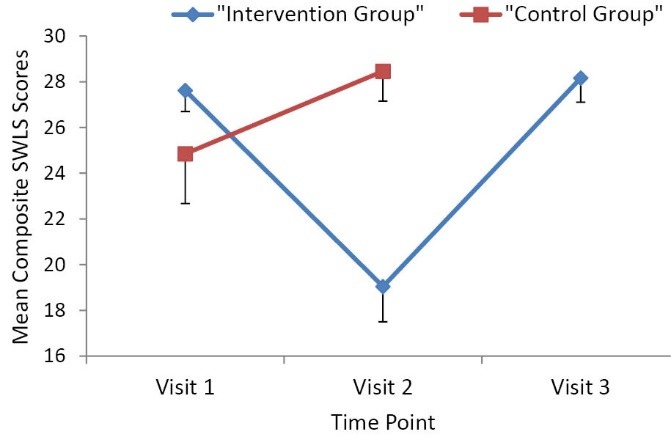
Intervention group vs. control group composite SWLS scores over time.
Standard deviations are included as the error bars.

Table 2 reports the mean SWLS scores by time period. The split-plot ANOVA demonstrated a statistically significant time x group interaction effect for SWLS scores (*F* = 32.75, *P *< 0.001). Mean ± standard error (SE) SWLS scores were significantly lower after the 1-week sedentary behavior-inducing intervention (19.0 [SD = 1.54], *P *< 0.001) compared to scores from before the intervention (27.62 [SD = 0.92]). Mean scores increased above baseline following the 1-week of resumption to normal physical activity (28.16 [SD = 1.05], *P *< 0.001). These findings suggest that a 1-week sedentary-inducing intervention detrimentally influenced quality of life (in an active sample of young adults), with SWLS scores returning back to baseline values after participants resumed normal physical activity patterns. Mean results are demonstrated graphically as compared to the control group in Figure 2. The mean scores for baseline SWLS in the intervention and control groups were not statistically, significantly different (27.62 vs. 24.85, *P *= 0.18). In sum, these findings suggest that a 1-week sedentary-inducing intervention among active individuals has a negative effect on life satisfaction at both group and individual levels.

In the intervention group, a mean absolute (Visit 2 minus Visit 1) change of -8.58 (95% CI: -5.91 – -11.24) for SWLS scores was observed. The relative percentage change (Visit 2 minus Visit 1/Visit 1) was -31.1%. The calculated Ƞ^2^_p_ value for SWLS scores was 0.469, which suggests that 46.9% of the variance for changes in life satisfaction may be accounted for by group assignment. The calculated ω^2^_p_ value for SWLS scores was 0.448. Additionally, Cohen’s d values were calculated to assess mean difference effect sizes, both between and within groups. The calculated Cohen’s d comparing the mean SWLS scores between the intervention and control groups at Visit 2 (post sedentary-behavior intervention) was 1.46. The calculated Cohen’s d_z_ (within-group) for the intervention group’s mean SWLS scores was 1.30 for Visit 1 vs. Visit 2 and was 1.28 for Visit 2 vs. Visit 3. The calculated Cohen’s d_z_ for the control group’s mean SWLS scores was 0.56 for Visit 1 vs. Visit 2. Lastly, and although there were no differences in baseline physical activity (self-reported or accelerometer-assessed) between the intervention and control groups, it is plausible to suggest that baseline physical activity may moderate the intervention effects. For example, highly active individuals may have a greater change in life satisfaction following a sedentary intervention than minimally active (i.e., meeting minimum physical activity guidelines) individuals. Sensitivity analysis did not, however, suggest such an effect, as determined by a visual plot (data not shown) of baseline physical activity and SWLS changes scores (Visit 2 minus Visit 1). Further, in a linear regression model, baseline accelerometer-determined steps were not associated with this SWLS change score in either the intervention (β = 0.122; 95% CI: -21.25 – -1.48; *p*=0.276) or control group (β = -0.317; 95% CI: -1.29 – 17.21; *P *= 0.145).

## Discussion

Our results are in accordance with our original hypothesis that a sedentary behavior inducing intervention would have detrimental effects on life satisfaction in active, young adults. As reported within the Results section, partial eta-squared and partial omega-squared values >0.14, as well as the Cohen’s d values (within the intervention group) >0.80 demonstrate that the magnitudes of our observed effects are large.^22^ The short duration of our intervention lends some support to the idea of weekly sedentary behavior having a bottom-up influence on life satisfaction and SWB.

The relationship between physical activity and life satisfaction lacks one profound mechanism to explain the association. Due to life satisfaction being a global health outcome (especially as assessed via the broad statements in the SWLS) where each individual consciously chooses which life aspects to weigh and how much influence they feel each aspect has on their overall satisfaction,^20^ it is plausible to believe that there are numerous mechanisms that synergistically mediate the physical activity-life satisfaction relationship. As mentioned previously, affect, physical self-worth, self-efficacy, and mental health have all been suggested to mediate this relationship.^11,12^ The emerging adulthood years (i.e., 18-25; ages included in our study) are characterized by increased negative self-evaluations and affective lability when compared to later life stages (e.g., midlife or older adulthood).^23,24^ This may put the age group in our study at an increased risk for some of the negative sedentary behavior-inducing mediating mechanisms (mentioned previously) that ultimately may have led to their decreased satisfaction with life scores. Given that physical activity has been positively associated with improved mental health (e.g., decreased depression symptomology),^25^ it is plausible to speculate that removing physical activity would have the opposite effect on these variables (and thus an indirect negative influence on life satisfaction).

Additional theories that may help explain the resulting decreases in life satisfaction are the activity theory and the need theory. The *activity theory* postulates that life satisfaction is determined by both the frequency of engagement in various activities as well as the degree of intimacy associated with these activities.^26^ Participants in our study were required to reduce the frequency of physical activity by eliminating exercise for an entire week. For any individuals in our study who potentially believe their sense of self-worth or general well-being is intimately associated with their weekly exercise routines and personal fitness levels, this reduction likely was especially difficult and detrimental to their life satisfaction. It is also possible that the intimacy associated with physical activity was in part attributed to various social interactions the individual engaged in while exercising (e.g., taking a group exercise class or exercising in the fitness center with a friend). For those who associate exercise with positive human interaction, removing physical activity may have thus reduced their level of social intimacy to some extent, potentially resulting in lower life satisfaction. The *need theory* states that life satisfaction is mainly regulated by an individual’s ability to satisfy his or her biological and psychological needs.^27^ It is possible that participants in our study have needs they use physical activity to help meet (e.g., the use of physical activity to attenuate existing symptoms of anxiety or attention deficit disorders, the participation in physical activity to maintain or hypertrophy existing muscle mass, or the participation in exercise to increase one’s self-esteem). By removing physical activity, it is possible that these individual needs were not met as well, resulting in decreased life satisfaction.

A limitation of this study is the utilization of a nonprobability convenience based sampling approach likely resulting in some degree of selection and sampling biases. Sampling bias can compromise the external validity of a study by reducing the ability of the study to be generalized to the rest of the population, whereas selection bias can lead to lower levels of internal validity for any observed differences or similarities within the samples. Another limitation of our study is that we utilized accelerometer derived step counts as our baseline step counts and pedometer derived step counts were used for the subsequent visit(s). However, we applied a correction factor based off a well-known comparison study by Tudor-Locke et al to take into consideration the fact that accelerometers tend to generate higher step counts than pedometers.^28^ The reduction in average steps/day from baseline to Visit 1 in the intervention group remained statistically significant with the *P* value changing from *P *< 0.001 to *P *= 0.0005. Additionally, the control group actually significantly increased their average daily steps, both without and with the applied correction factor (*P* value changed from *P *= 0.03 to *P *= 0.001). The step count comparisons are displayed in Table 1. The pedometer used in the 2002 Tudor-Locke et al study^28^ was the Yamax Digi-Walk 200 pedometer (i.e., the same pedometer used in our current study). The GT9X accelerometer by ActiGraph is the newest model of the 7164 ActiGraph accelerometer used for comparison in the 2002 study. In 2010, a study compared the 7164 accelerometer with three versions of the ActiGraph GT1M accelerometer, finding no statistically significant differences in their outputs.^29^ In 2012, a comparison study found good agreement between the ActiGraph GT1M and the ActiGraph GT3X, which is the most recent model prior to the current ActiGraph model, the GT9X.^30^ Comparison studies for the GT3X and the GT9X have not yet (to our knowledge) been published. Taken together, these unadjusted and adjusted findings suggest that the different instrument (accelerometer to measure steps vs. pedometer to measure steps) used at baseline compared to the subsequent weeks did not appreciably influence our experimental findings. Major strengths of this study include the utilization of an experimental design to manipulate sedentary behavior and the use of both objective and subjective measures of physical activity to confirm study inclusion criteria.

## Conclusion

The present findings suggest that a 1-week sedentary behavior inducing intervention has a statistically significant, negative effect on life satisfaction. As mentioned previously, this is the first randomized controlled intervention (to our knowledge) to experimentally increase sedentary behavior and evaluate this outcome in a free-living setting. Coupled with known information regarding the benefits of physical activity on life satisfaction, the findings from this study provide evidence for a cause-and-effect relationship between sedentary behavior and life satisfaction in active individuals. Our observation that the intervention group (who decreased their physical activity) had unfavorable changes in life satisfaction, coupled with our observation that the control group (whom inadvertently increased their physical activity) had favorable changes in life satisfaction, supports this cause-and-effect relationship and highlights the powerful role of physical activity and sedentary behavior on this health outcome. These findings underscore the importance of maintaining a normal routine of physical activity to avoid decreases in life satisfaction in the young adult population. Clinicians and counselors who work with sedentary patients suffering from low levels of SWB and poor life satisfaction may recommend beginning a physical activity routine to improve these negative self-rated health outcomes.

Future studies looking to build off of these observations may consider recruiting only highly active individuals (for instance, individuals who exercise 4-5 days a week and accumulate at least 300 min/wk of MVPA) as compared to those who solely meet minimum MVPA guidelines, as this may help to confirm our findings which did not suggest a moderating role of baseline physical activity levels on changes in life satisfaction. Additionally, employing a mixed study design that utilizes some form of qualitative assessment of the sedentary intervention (e.g., focus groups or interviews with participants asking how the sedentary behavior intervention affected them) may help to elaborate further on the potential mechanisms that mediate the relationship between sedentary behavior and life satisfaction. This qualitative assessment could also address individual’s definitions of life satisfaction; it would have been informative to evaluate and compare how participants in the present study would have ranked physical activity participation alongside other reported components of their overall life satisfaction.

## Implications for policy and practice

The field of health promotion encompasses all aspects of wellness. While impressive efforts have been made to advocate the importance of regular physical activity, there remains a pervasive need for better education regarding the deleterious consequences of excessive sedentariness. This includes a need for the adoption and promotion of formal guidelines that include minimizing sedentary behavior. This study was able to experimentally manipulate sedentary behavior time, demonstrating that decreased overall physical activity levels and the removal of regular structured exercise (resulting in increased sedentariness) were related with significant impairments in perceived life satisfaction. It is the responsibility of health promotion professionals to develop policy and practices that ultimately help to improve one’s quality and satisfaction with life. Health promotion professionals should consider it equally as important to inform others about the dangers of sedentary behavior as they do about the benefits of physical activity. For example, health promotion professionals working with those who have somewhat recently adopted a physical activity routine could use the findings of this study to provide motivation for sustaining this new level of activity and not relapsing to a more sedentary lifestyle.

## Ethics approval

All participants provided written, informed consent prior to any data collection activities. This study was approved by the author’s institutional review board.

## Competing interests

The authors are not aware of any affiliations, financial support or memberships that may influence this manuscript; thus, we declare no competing interests.

## Funding

No funding was used to conduct this study or to prepare this manuscript.

## Authors’ contributions

Both authors had substantial contributions to the conception/design of the study and data analysis. ME drafted the manuscript; PL served as corresponding author and contributed evaluations/revisions of the manuscript. ME performed the data collection. Both authors approve the current version of this manuscript and agree to be accountable for all aspects of this work.

## Acknowledgments

The authors indicate that no other individuals have contributed to this work.

**Table 1:** Demographic characteristics of the analyzed sample (N = 39)

**Demographic variable**	**Intervention group (n = 26)**		**Control group (n = 13)**		***P*** ** value†**
	**n**	**Mean (SD)/%**	**n**	**Mean (SD)/%**	
Gender, %					0.65
Male	10	38%	6	46%	
Female	16	62%	7	54%	
Race-ethnicity, %					0.65
Mexican American	1	4%	0	0%	
Non-Hispanic White	16	61.5%	9	69%	
Non-Hispanic Black	7	27%	4	31%	
Other/multi-race	2	7.5%	0	0%	
Education Status, %					0.31
Undergraduate	20	77%	8	62%	
Graduate	6	23%	5	38%	
BMI, kg/m^2^	26	25.35 (6.82)	13	26.13 (3.67)	0.71
Age, years	26	21.69 (2.71)	13	22.08 (2.75)	0.69
Baseline MVPA, min/wk (IPAQ-SF)	26	423.85 (206.03)	13	577.50 (293.21)	0.07
Baseline MVPA, min/wk (Accelerometer)	26	317.65 (111.56)	13	362.29 (145.24)	0.30

Abbreviation: BMI, body mass index; MVPA, moderate-to-vigorous physical activity; IPAQ-SF, International Physical Activity Questionnaire, Short-Form. ^a^ An independent sample student *t* test was used to calculate differences for the continuous variables across the two groups (intervention vs. control). For the categorical variables, a chi-square analysis was used to calculate differences for the categorical variables across the two groups.

**Table 2:** Mean changes in SWLS scores across the study time period (N = 39)

**SWLS scores/group**	**Visit 1 (Week 1)**	**Visit 2 (Week 2)**	**Visit 3 (Week 3)**	***F*** ** value**	***P*** ** value (Visit 1**→**2)**	***P*** ** value (Visit 2**→**3)**
Intervention	27.62 (0.92)	19.04 (1.54)	28.16 (1.05)		<0.001	<0.001
Control	24.85 (2.18)	28.46 (1.30)	-			

Abbreviation‏: SWLS, Satisfaction With Life Scale. Standard error for each mean score are reported after the mean in parenthesis. *P* values for Visit 1→2 were calculated using repeated measures ANOVA and *P* values for Visit 2→3 were calculated using paired *t* tests. The reported *P* value and *F* value are from the split-plot ANOVA are from the group*time interaction.
